# Supplementary material for: Taurine Enhances Iron-Related Proteins and Reduces Lipid Peroxidation in Differentiated C2C12 Myotubes
Source: Antioxidants (Basel). 2020 Oct 31;9(11):1071. doi: 10.3390/antiox9111071 (PMC7693586; doi:10.3390/antiox9111071)
Supplement: Supplementary file 1 [file antioxidants-09-01071-s001.pdf]

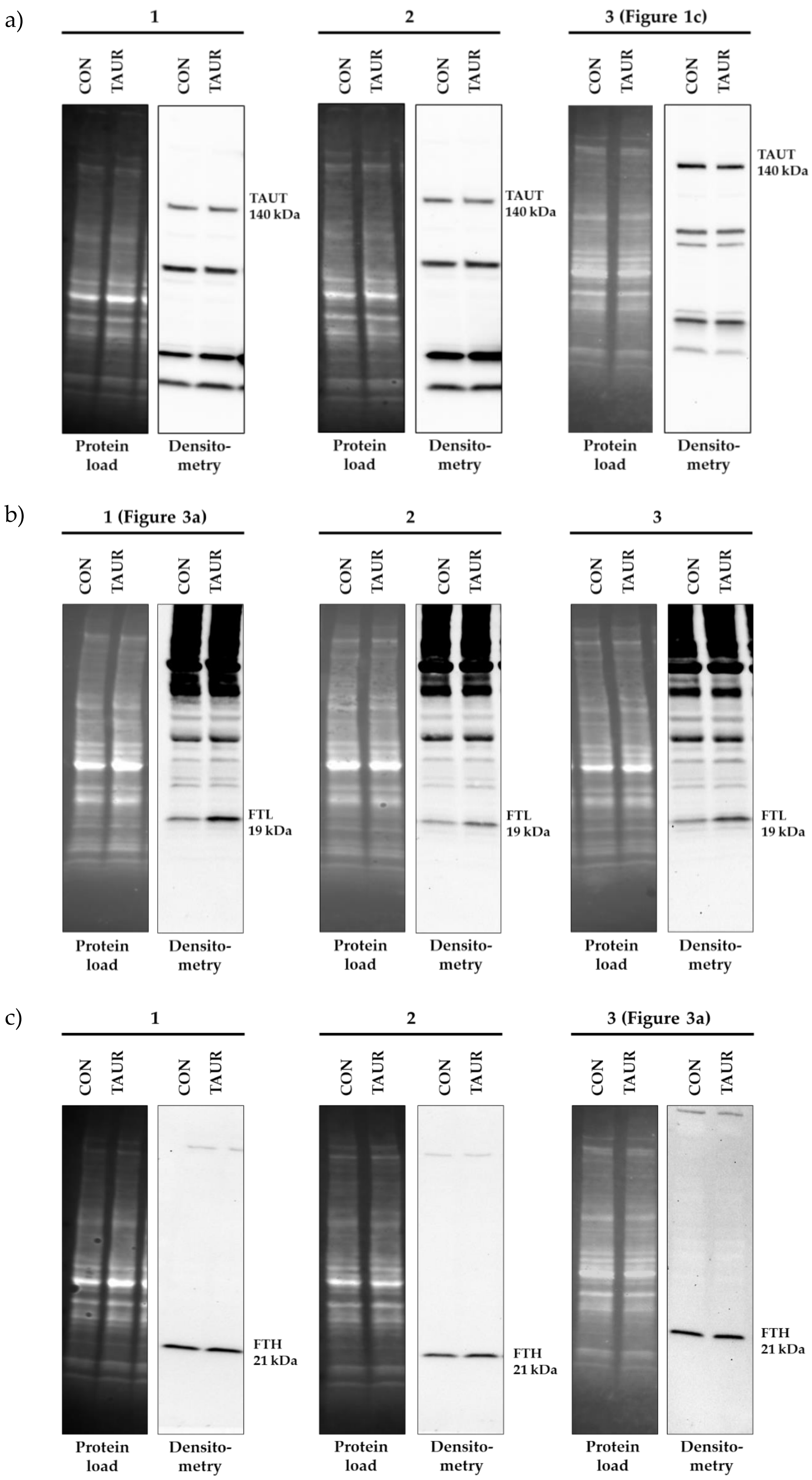

a)
1
2
3 (Figure 1c)
CON
TAUR
CON
TAUR
TAUT
140 kDa
Protein
load
Densito-
metry
b)
1 (Figure 3a)
2
3
CON
TAUR
CON
TAUR
FTL
19 kDa
Protein
load
Densito-
metry
c)
1
2
3 (Figure 3a)
CON
TAUR
CON
TAUR
FTH
21 kDa
Protein
load
Densito-
metry

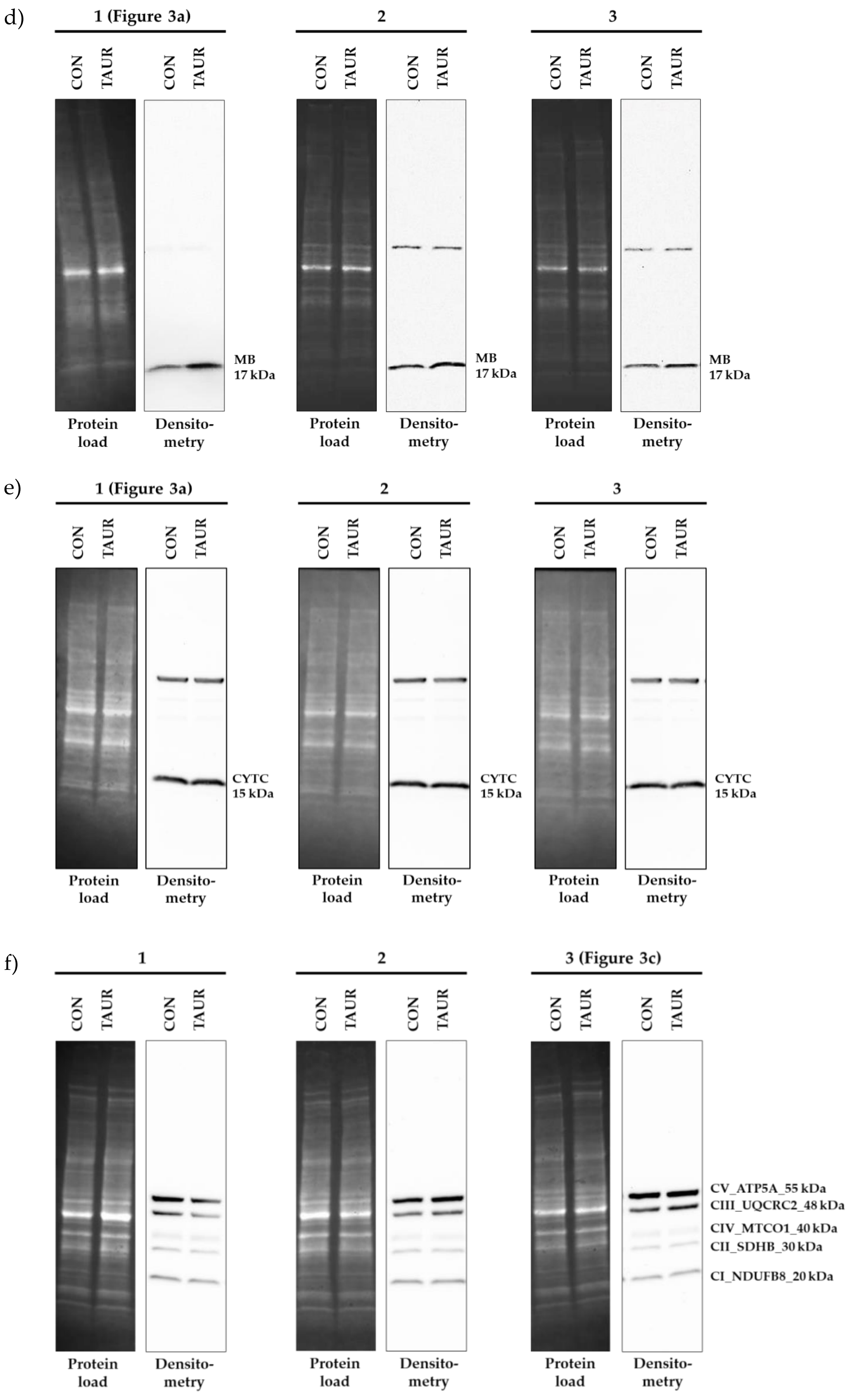

S1:Western blots of three independent experiments showing the protein load of the membrane and the respective target bands of the a) taurine transporter (TAUT) at 140 kDa, b) ferritin light chain (FTL) at 19 kDa, c) ferritin heavy chain (FTH) at 21 kDa, d) myoglobin (MB) at 17 kDa, e) Cytochrom C (CYTC) at 15 kDa and f) subunits of mitochondrial oxidative phosphorylation (OXPHOS) complexes CI (NADH dehydrogenese), CII (succinate dehydrogenase), CIII (ubiquinol-cytochrome c reductase), CIV (cytochrome c oxidase) and CV (ATP-Synthase) in response to control medium- (CON) and taurine- (TAUR) treatment
